# The Action of Strychnine on the Cerebrum

**Published:** 1892-02

**Authors:** 


### THE ACTION OF STRYCHNINE ON THE CEREBRUM.

The majority of authorities attribute the action of strychnine to its influence on the spinal cord and medulla oblongata. These conclusions are warranted not only by the character of the symptoms produced in strychnine-poisoning, but also by the fact that after fatal poisoning by strychnine, large amounts of the alkaloid are to be found in these localities, while but a trace is detectable in the cerebrum. In addition, there are no direct experiments as to the action of strychnine on the cerebrum, and but few observations have been attempted in this connection, and they are mostly of a negative character.

In the Centralblatt fur Klinische Medicin, No. 36, 1891, is an account of some experiments by Dr. Biernacki, who has tested the action of strychnine on the cerebrum by exposing the motor centers of the cortex, determining the weakest possible stimulus which would produce a motor reaction, then injecting strychnine and testing the changes in irritability. His results show that strychnine produces marked reduction in the irritability of the cortex, whether administered subcutaneously or locally applied. It is not clear, however, as to whether strychnine has a direct influence on the gray substance of the cortex or not, since it is possible that the apparent reduction in irritability may be due to the action of this alkaloid on other parts of the central nervous system.—*Therapeutic Gazette.*

